# Marathon penguins – Reasons and consequences of long-range dispersal in Fiordland penguins / Tawaki during the pre-moult period

**DOI:** 10.1371/journal.pone.0198688

**Published:** 2018-08-29

**Authors:** Thomas Mattern, Klemens Pütz, Pablo Garcia-Borboroglu, Ursula Ellenberg, David M. Houston, Robin Long, Benno Lüthi, Philip J. Seddon

**Affiliations:** 1 Department of Zoology, University of Otago, Dunedin, New Zealand; 2 Global Penguin Society, Marcos Zar 2716, Puerto Madryn (9120), Chubut, Argentina; 3 Antarctic Research Trust, Am Oste-Hamme-Kanal 10, Bremervörde, Germany; 4 Centro Nacional Patagónico (CONICET), Boulevard Brown 2825, Puerto Madryn, Chubut, Argentina; 5 Department of Ecology, Environment and Evolution, La Trobe University, Melbourne, Australia; 6 Science and Policy Group, Department of Conservation, Auckland, New Zealand; 7 West Coast Penguin Trust, Hokitika, New Zealand; 8 Antarctic Research Trust, c/o Zoo Zürich, Zürichbergstr, 221, Zürich, Switzerland; Phillip Island Nature Parks, AUSTRALIA

## Abstract

Migratory species often roam vast distances bringing them into contact with diverse conditions and threats that could play significant roles in their population dynamics. This is especially true if long-range travels occur within crucial stages of a species’ annual life-cycle. Crested penguins, for example, usually disperse over several hundreds of kilometres after completing the energetically demanding breeding season and in preparation for the costly annual moult. A basic understanding of crested penguins’ pre-moult dispersal is therefore paramount in order to be able to assess factors affecting individual survival. The Fiordland penguin, or Tawaki, the only crested penguin species breeding on the New Zealand mainland, is currently one of the least studied and rarest penguin species in the world. We successfully satellite tracked the pre-moult dispersal of 17 adult Tawaki from a single colony located in the species’ northern breeding distribution. Over the course of 8–10 weeks the penguins travelled up to 2,500 km away from their breeding colony, covering total swimming distances of up to 6,800 km. During outbound travels all penguins headed south-west within a well-defined corridor before branching out towards two general trip destinations. Birds leaving in late November travelled towards the Subtropical Front some 800 km south of Tasmania, whereas penguins that left in December headed further towards the subantarctic front. Using K-select analysis we examined the influence of oceanographic factors on the penguins’ dispersal. Water depth, surface current velocity and sea level anomalies had the greatest influence on penguin movements at the subantarctic Front, while sea surface temperature and chlorophyll a concentration were key for birds travelling to the subtropical front. We discuss our findings in the light of anthropogenic activities (or lack thereof) in the regions visited by the penguins as well as the potential consequences of Tawaki pre-moult dispersal for the species’ breeding distribution on the New Zealand mainland.

## Introduction

Conservation of migratory animal species is a daunting task. Unlike sedentary species that principally utilize spatially definable habitats which, at least in theory, allows for a holistic conservation approach, migratory species often roam vast distances, traversing or entering regions with differing conservation challenges [1,2]. While many migratory land bird species can be monitored with comparatively simple measures along their flyways and in their breeding, stop-over, or over-wintering destinations [3], the journeys of migratory seabirds with open-ocean destinations can be studied only with technological assistance [4]. At least some basic knowledge about such species’ whereabouts outside the breeding period is vital to be able to put population trends into a valid ecological context [5]. This is particularly true when it comes to the interpretation of survival rates and the assessment of factors influencing population dynamics [6]. Crested penguins from the New Zealand region provide a good example of this challenge.

New Zealand hosts one third of the world’s penguin species within its Exclusive Economic Zone [7]. Of these, four belong to the genus of crested penguins (*Eudyptes*), three of which are endemic to New Zealand, namely the Erect-crested penguin (*E*. *sclateri*), the Snares penguin (*E*. *robustus*) and the Fiordland penguin / Tawaki (*E*. *pachyrhynchus*). (Note: For brevity and clarity, throughout this paper we will refer to the latter species only as *Tawaki*, its native and legal name in New Zealand.) Populations of both, Erect-crested and Tawaki, are believed to be undergoing significant declines [8]. The reason for these declines are unclear, though ocean warming and associated changes in marine productivity are suspected to play a role [9]. While the Erect-crested and Snares penguins occur only on subantarctic islands, Tawaki is the only crested penguin species to breed on the New Zealand mainland, where it is exposed to a variety of threats ranging from introduced predators to human disturbance [10]. Unlike its subantarctic congeners, Tawaki are subject to frequent monitoring efforts so that any substantial negative changes in population size can be met with conservation actions. However, in order to succeed, such actions require at least a basic understanding of factors responsible for any population changes. This is especially vital in times of limited resources for conservation [11,12].

Populations of long-lived vertebrates, like seabirds, are most sensitive to changes in adult survival [13]. In penguins, the pre-moult phase is crucial for adult survival [14]. Chick rearing duties generally result in substantial loss of body mass which needs to be replenished rapidly in preparation for the energy demanding annual moult [15]. During the breeding season, Tawaki lose up to 50% of their pre-breeding weight which they have to fully restore on their annual 60–80 day pre-moult foraging trips between December and February [10]. As penguins have to remain on land for at least three weeks to complete the moult [16], it is important for them to be able to access abundant food resources during the pre-moult period. Breeding sites are devoid of penguins at this stage so that it can be assumed the birds have offshore foraging destinations. It is conceivable that the penguins during this time visit regions subject to large scale industrial fisheries which can have profound impacts on survival rates of other top-level predators [17]. Hence, interpretations of annual survival rates must consider the species’ exposure to sea-based threats and therefore require knowledge of their pre-moult dispersal.

We studied the pre-moult journeys of Tawaki after the conclusion of the 2016 breeding season at one of the species’ core breeding sites on the southern Westcoast of the South Island, New Zealand. Our goal was to locate the ocean regions the birds visit during their dispersal period, and thus to identify the environmental variables associated with the penguins’ selected dispersal trajectories.

## Material and methods

### Ethics statement

The research was carried out with permission from the New Zealand Department of Conservation (permit number RES-38882). Ethics approval was obtained from the University of Otago’s Animal Ethics Committee (AEC-04/14).

### Study species & site

**Fig 1:**
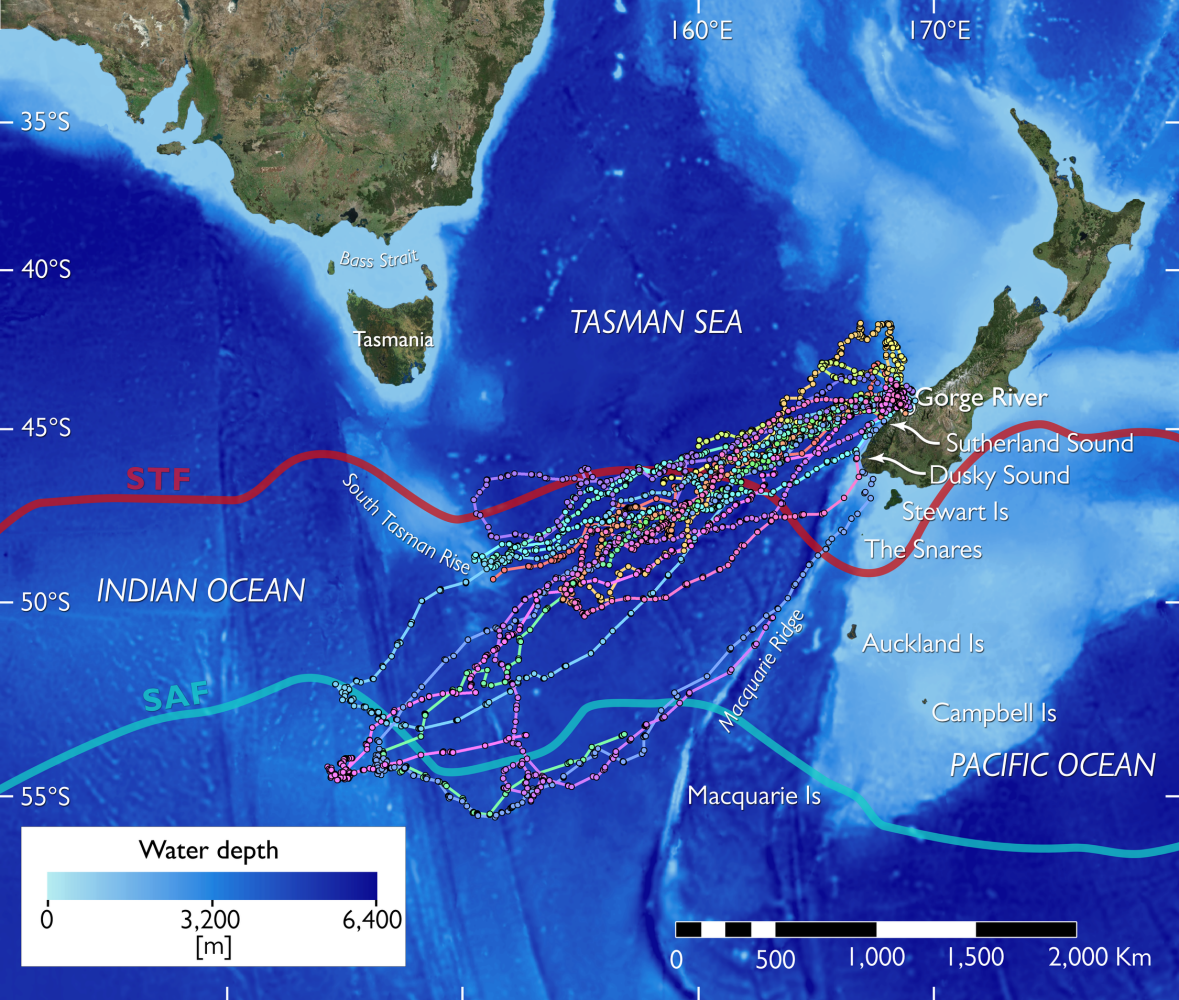
Overview of the southern tasman sea bathymetry and important geographic and oceanographic features. The main oceanic fronts are indicated as red (Subtropical Front, STF) and light blue lines (Subantarctic Front, SAF). Traveling paths of 17 tawaki from Gorge River, South Westland, New Zealand, during their pre-moult journey of between November 2016 and March 2017 are shown as coloured dots and lines. Each colour represents a different individual. Tracks were derived from filtered satellite data before daily averaging; see methods for details. Note that only 5 complete tracks–i.e. tracks where the birds returned to land to moult before the satellite transmitters stopped working–were recorded.

With an estimated population size of 5,500–7,000 mature birds the Fiordland penguin / Tawaki (*Eudyptes pachyrhynchus*) is the third-rarest penguin species world-wide and is classified under the IUCN Red List criteria as ‘Vulnerable’ [18]. It is endemic to the south-western ranges of New Zealand’s South Island (southern West Coast and Fiordland) as well Stewart Island and its outliers [10] (Fig 1). In 2014, a long-term study was launched to investigate the species’ marine ecology (‘The Tawaki Project’, http://www.tawaki-project.org) over its entire breeding range [19].

We investigated pre-moult dispersal of penguins breeding along a remote stretch of coastline south of Gorge River, South Westland (S44.188, E168.188). The site is located approximately 30 km from the nearest human settlement, Jackson Bay on the West Coast of New Zealand’s South Island (Fig 1). Tawaki live and breed in loose colonies in primary forest along the coast. The current estimate of the population size for the region (Cascade River, S44.029, E168.374, to Martins Bay, S44.335, E167.997) is around 870 breeding pairs [20], which represents nearly one third of the species’ global population.

### Satellite telemetry

We deployed Sirtrack Kiwisat 202 satellite transmitters (LxWxH: 60x27x17mm; weight: 32g) on adult penguins to track their at sea movements between the end of their breeding season (August-November) and their annual moult (February-March) [16]. Between 11 and 13 November 2016, a total of 20 birds, 12 males and 8 females determined from body weights and bill dimensions [16], was fitted with devices using black adhesive tape (Tesa 4651, Beiersdorf AG, Germany) following the methods described in [21]. In addition to tape, rubber glue (Pattex Classic, Henkel AG, Germany) was applied to the device base to provide additional bond. After attachment with tape, the top surface of the wrapped device was sealed with 2-component epoxy glue (Weicon Epoxy Minute Adhesive, Weicon GmbH & Co. KG, Germany) to prevent the tape from loosening over time and to provide a smooth finish to enhance hydrodynamics. Battery life of the devices was expected to be 6 months and, therefore, sufficient to cover entire pre-moult trips which were expected to last between 8 and 12 weeks [10].

### Argos data processing

A total of 3,989 positions were recorded for 19 birds; one device failed to transmit any location information. Moreover, signals from two birds stopped before they initiated their pre-moult trip, most likely due to technical failures or device loss. The data from the remaining 17 birds was filtered to remove short-term foraging trips performed by the birds before their final departure, so that 2,827 positions remained. Of these, 1,384 positions (48.9%) did not have an accuracy estimation (ARGOS location classes ‘A’ and ‘B’), so that simple data filtering by location classes only [22] would have unduly reduced the numbers of positions obtained. Instead, the Speed-Distance-Angle-filter algorithm [23] was applied in R [24] using the corresponding package ‘argosfilter’ [25]. The ‘sdafilter’ algorithm is based on the traveling speed of the tracked animal, distance between successive locations, as well as turning angle, and requires the specification of a maximum traveling speed threshold [23]. We used a threshold of 2.0 m/s based on the mean of penguin swimming speeds as reported in [26]. After applying the filter, 2,326 positions (82.3%) were retained for further analysis. As satellite tags often transmitted several positions per day, we calculated daily means of both time-of-the-day per positions and location. For each bird, mean time-of-the-day was calculated by converting date and time of fixes recorded on a given calendar day (UTC time code) into UNIX timestamps (i.e. number of seconds elapsed since midnight 1 January 1970), calculating the mean for these timestamps, and back-converting the result to date-time format. Averaging of locations was achieved by using ‘geomean’ (R package ‘geosphere’; [27]) on daily fixes. Thus, only a single mean time and position per day and individual remained for subsequent analysis. ‘Daily travel distances’ and ‘average travel speeds’ were then determined using the great-circle-distance between consecutive mean daily positions (function ‘distCosine’ from ‘geosphere’) and the corresponding time differences.

### Spatial data analysis

Basic spatial analysis was performed in ArcGIS [28] using filtered daily positions. Firstly, distances were calculated between consecutive points of each bird’s filtered data set. Trip length was calculated as cumulative sum of these distances. The position furthest away from the location of device deployment was defined as ‘maximum range’.

Filtered satellite locations were used to compute kernel density distributions. To account for incomplete data sets, two different density distributions were calculated, namely kernels of the outward (i.e. movement away from the breeding colony) and, conversely, the inward-bound portions of recorded trips. For the outward-bound kernels, satellite positions recorded between the day of departure and the day that birds reversed their travel trajectory (‘trip reversal date’) were used. Data from birds for which no trip reversal date could be determined were excluded from the kernel analysis. Similarly, inward-bound kernels were determined from satellite positions recorded between the trip reversal date and the day the penguins made landfall; only data from birds that reached mainland waters (<5 km from coast) were included in the analysis. 20, 40, 60 and 80% quantile kernel densities were calculated using the ‘Geospatial Modelling Environment’ [29].

To assess relative rates of travel throughout the penguins’ trips, daily travel distances were calculated for birds where complete data sets were recorded. To account for the variable trip lengths, absolute time (i.e. days) was transformed to relative trip time (i.e. % of trip duration) for each bird. A Generalized Additive Mixed Model [30] was developed to assess changes in rates of travel rates using daily distance as response variable, relative trip time as factor and penguin id as random effect (analysis carried out in R using package ‘mgcv’ [31]).

### Environmental data

Foraging movements were plotted against selected oceanographic variables to assess their influence on the penguins. Oceanographic data were derived from the following sources. Bathymetry data was obtained as 250m gridded data from NIWA, Wellington, NZ (https://www.niwa.co.nz/our-science/oceans/bathymetry/download-the-data). Sea Surface Temperature (SST) and Chlorophyll a (CHL A) concentration measured at 4km resolution from Nasa’s AquaMODIS program were downloaded from https://oceancolor.gsfc.nasa.gov/, OSCAR third degree resolution ocean surface currents were accessed via https://podaac.jpl.nasa.gov/dataset/OSCAR_L4_OC_third-deg, and Sea Level Anomalies (SLA) were acquired from https://podaac.jpl.nasa.gov/dataset/SEA_SURFACE_HEIGHT_ALT_INTERIM_GRIDS_L4_2SATS_5DAY_6THDEG_V_JPL1609. All data sets were obtained as rolling 32-day composites encompassing the period from 18 December 2016 to 18 January 2017, except for SLA which is only available as 5-day composites. The seven SLA data sets available for the date range 15 December 2016 to 19 January 2017 were averaged using the ‘Raster Calculator’ tool in in ArcGIS [28] to match the temporal resolution of the other data sets. Finally, Mixed Layer Depth (MLD) data available as 0.5x0.5° gridded data was obtained from the CSRIO Atlas of Regional Seas, which is a modelled data set based on various oceanographic profile data collected over the past 50 years [32].

For the subsequent habitat selection analysis, all environmental data were reprocessed to a matching grid with a 57.4 km cell size representing the lowest common spatial resolution of all data sets, i.e. based on the MLD data set. All data sets were resampled using the ‘Resample Raster’ processing function in ArcGIS.

### Habitat selection at journey destination

To determine the environmental variables that best describe the ocean regions targeted by the penguins during the pre-moult dispersal period, we conducted a K-select habitat selection analysis [33]. For this we used only satellite data for the nine birds for which the trip reversal date was recorded. Satellite fixes representing the 10 days before and after each bird’s trip reversal date were extracted and used for the subsequent analysis. Individual habitat selection was assessed using marginality that describes the difference between the mean environmental conditions at each penguin’s trip destination, and the mean conditions sought out by each bird based on their movements while at their destination. K-select analysis involves a PCA on the marginality vectors of each animal and extracts the relevant part of the individual habitat selection. If all animals exhibited the same patterns of habitat selection, all marginality vectors would be oriented in the same direction. For an in-depth description of this methods and its mathematical derivation refer to [33].

Basic statistical analyses were carried out in R [24]. Linear mixed-effects models were conducted using the package ‘nlme’ [34], K-select analysis was performed in R using the package ‘adehabitat’ [35].

## Results

A total of 20 penguins were handled for transmitter deployment of which 17 were weighed. Mean weight of all birds was 3,162±395 g (n = 18). Males were generally heavier (mean weight: 3,415±298 g, n = 10) than females (2,800±171g, n = 7).

### Basic trip parameters

Satellite transmitter deployments on 19 birds yielded valid data; between 1 and 8 positions were recorded per day (Fig 1). Location averaging brought the total fix number to 681 daily fixes. Seventeen birds commenced pre-moult trips between 12 November and 18 December 2016 (median date: 2 December 2016; Table 1). Nine birds were tracked until they initiated their return journey. The trip reversal dates ranged from 27 December 2016 to 26 January 2017 (median date: 6 January 2017), between 23 and 42 days (median: 32 days, n = 9) after the start of the journey. By then the birds had distanced themselves a median 1,973 km (range: 1,371–2,440 km, n = 9) from their origin (Fig 1). Complete trips could be determined for five birds, all of which required less time to return to the mainland (median: 29 days, range: 23–36 days, n = 5) when compared to the outward-bound portion of their journey (Table 1). Total swimming distance of completed trips ranged between 3,505 and 6,801 km (median: 5,381 km, n = 5).

**Table 1:** Overview of individual trip statistics of 17 adult Tawaki performing their pre-moult dispersal after completion of the breeding season 2016. Trip destinations (‘Trip Dest’) could be broadly distinguished as Subtropical Front (STF) and Subantarctic Front (SAF). Abbreviations for landfall locations are Gorge River (GR), Sutherland Sound (SS), and Dusky Sound (DS); see Fig 1 for an overview of tracks and locations. Travel speed (Daily travel distance) is provided as Median and range.

BirdID	Sex	Body mass [kg]	Trip Start	Trip End / Last Fix	Trip Duration [days]	Landfall location	Trip Length [km]	Max Range [km]	Trip Dest	Trip reversal	Outward journey [days]	Inbound journey [days]	Daily travel distance [km/day]	
													*median*	*range*
**Complete Trips**														
F45	female	2.50	18-12-16	22-02-17	66	GR	5,381	1,973	SAF	26.01.17	39	23	69.7	0.7–222.8
M49	male	3.60	05-12-16	20-02-17	77	GR	5,597	2,252	SAF	16.01.17	42	35	68.9	1.8–180.5
F50	female	2.90	19-11-16	01-02-17	74	GR	4,459	1,617	STF	27.12.16	38	36	64.4	4.2–178.8
M42	male	3.05	22-11-16	27-01-17	66	SS	3,505	1,371	STF	01.01.17	40	26	54.8	1.3–122.9
F48	female	2.95	03-12-16	08-02-17	67	DS	6,801	2,288	SAF	08.01.17	36	31	83.0	5.0–215.8
**Inbound journey incomplete**														
M44	male	3.40	13-12-16	02-02-17	51		4,870	2,440	SAF	24.01.17	42		69.9	1.0–222.8
M46	male	3.85	05-12-16	22-01-17	48		3,448	1,564	STF	28.12. 16	23		70.9	3.7–139.1
M52	male	3.95	01-12-16	18-01-17	48		4,244	2,245	SAF	06.01.17	36		78.4	3.1–225.4
F60	female	2.90	01-12-16	11-01-17	41		2,993	1,392	STF	27.12. 16	26		58.7	1.3–122.4
**Outward journey incomplete**														
F47	female	3.00	30-11-16	08-12-16	8		473	364					36.2	7.1–89.1
M51	male	3.20	01-12-16	28-12-16	27		1,798	1,594					61.3	2.3–134.9
F53	female	2.65	01-12-16	03-12-16	2		154	153					14.7	3.8–63.7
M54	male	3.45	06-12-16	26-12-16	20		1,420	1,167					47.9	3.4–131.7
M56	male	3.40	11-12-16	21-12-16	10		927	828					59.5	0.4–142
M58	male	3.00	04-12-16	19-12-16	15		1,121	888					62.3	1.0–172.8
M59	male	3.25	12-11-16	06-01-17	55		2,686	1,280					49.6	3.1–101.2
F61	female	2.95	11-12-16	01-01-17	21		2,057	1,635					76.2	2.0–177.3

### Kernel densities

**Fig 2:**
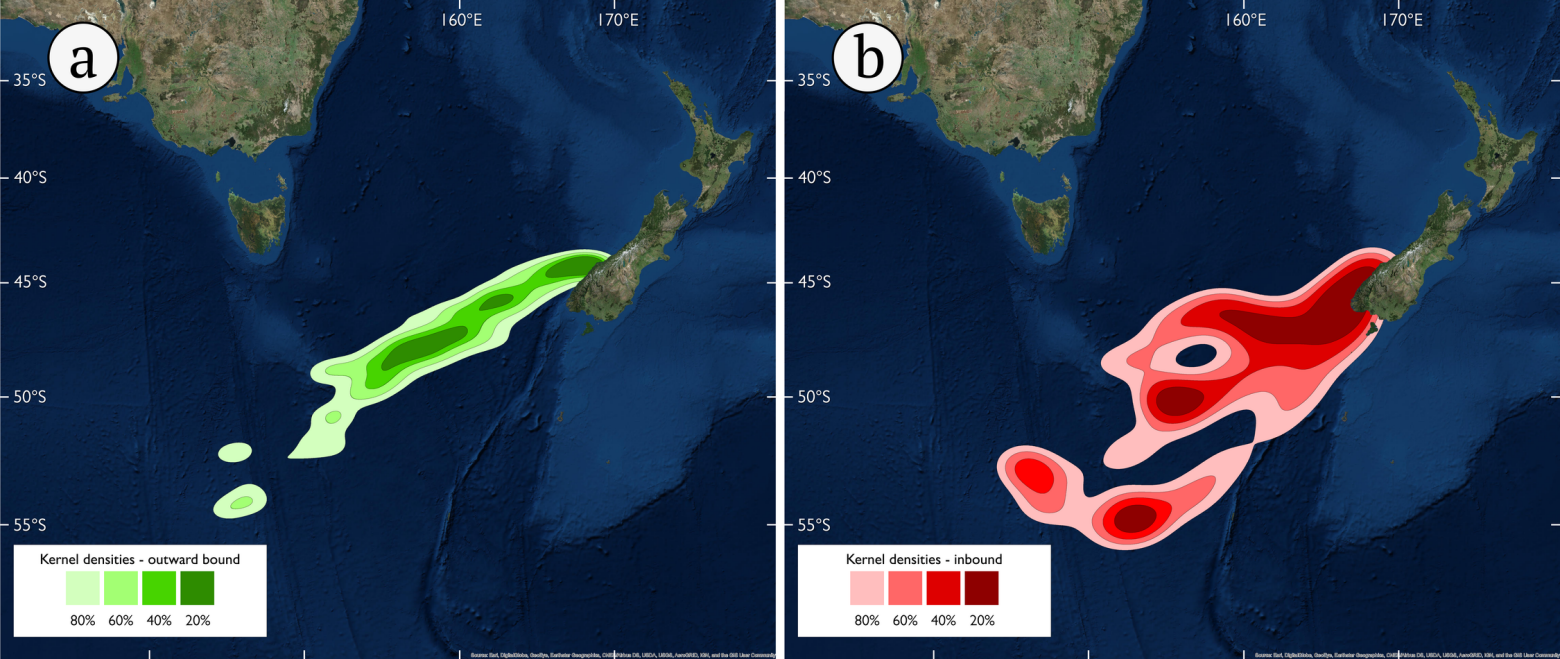
**Kernel densities of Tawaki positions during the outward-bound portion of their pre-moult journey (A) and their inbound journey (B).** Note that only complete data sets were used for calculation of kernels; see methods for details.

Nine birds could be satellite tracked until they initiated the return journey (Table 1). A total of 322 filtered, daily positions were used to calculate the outward-bound kernel densities, revealing consistent travelling trajectories. The majority of positions were recorded within a 300–400 km wide corridor (lateral spread of 80% kernel) that extended more than 2,000 km to the south-west of the New Zealand mainland (Fig 2A). The complete journey could be determined for five birds and resulted in 116 filtered daily positions to calculate inward-bound portion of their trips. At this stage, movement trajectories were far less consistent with positions being spread out more than 1,000 km (Fig 2B).

### Daily travel distances

**Fig 3:**
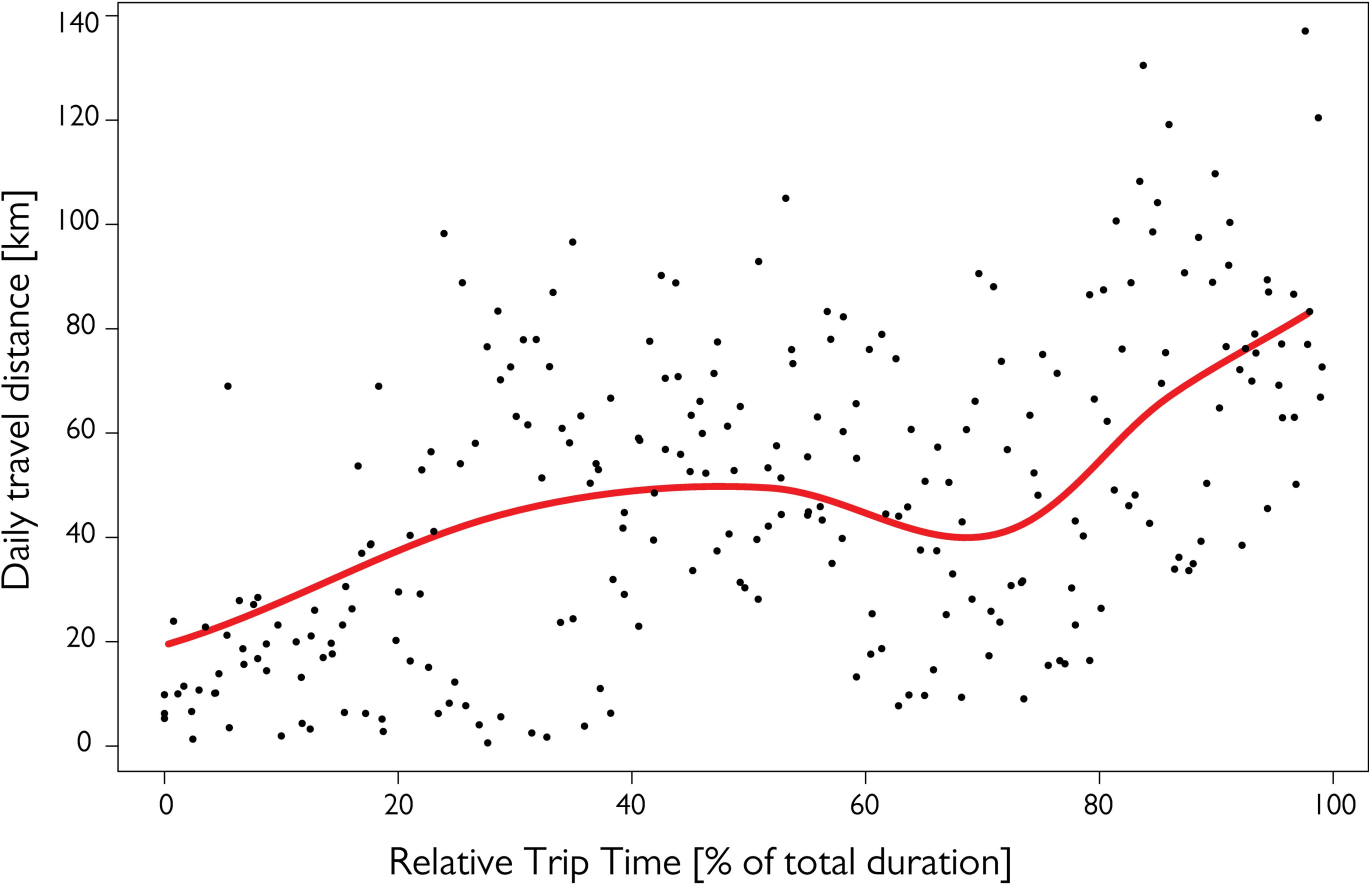
Daily travel distance over the course of pre-moult journeys of five Tawaki. Only data from birds (n = 5) completing their journey while satellite transmitters were still active were included. Due to differences in journey duration (range: 66–77 days, see Table 1) temporal distribution of daily travel distances (black dots) is plotted against the relative time of the trip. The red line indicates the fit of a Generalized Additive Mixed Model (see methods for details).

Daily travel distances indicate a steady increase during the first third of the penguins’ journey from about 20 km per day to an average 50 km per day (Fig 3). During the second third, distances travelled per day remained between 40 and 60 km per day, before the penguins increased their daily travel quota steadily to cover an average 80 km or more per day towards the end of their journeys.

### Trip destinations

Trip destinations can be broadly categorized into two regions, one south of the Subtropical Front (STF, n = 4 birds) and another just south of the Subantarctic Front (SAF, n = 5 birds) (Fig 1). Depending on the trip destination there were obvious differences in basic trip parameters (Table 1). Both maximum range and daily travel distance proved to be significantly different depending on whether birds foraged at the STF or travelled further on to the SAF (Table 2). Maximum ranges of birds foraging at the STF were nearly 750 km shorter when compared to birds visiting the SAF. Moreover, penguins that moved to the SAF tended to depart a week later and travelled more than 10 km further per day. The sex of the birds had no noticeable effects on trip parameters (Table 2).

**Table 2:** Linear mixed-effects models of the main trip parameters for nine Tawaki fitted with satellite transmitters during their pre-moult dispersal (December 2016 –February 2017). The base model uses trip destination (Subtropical Front, STF or Subantarctic Front, SAF) as well as sex as fixed effects, and BirdID as random effect. Note that only data from birds that completed the outward-bound portion of their journey before transmitters stopped working were included in the analysis. Also note that trip duration and total trip length could only be determined for five birds.

	PARAM ~ DESTINATION+SEX+(1|BIRDID)				
	Estimate	Std Error	DF	t	p
Departure Date [days]*					
Intercept	08-12-2016	4.77	6	8950.755	<0.001
Front (STF)	-11.3	5.34	6	-2.121	0.078
Sex (Male)	-0.7	5.34	6	-0.128	0.903
**Max Range [km]**					
Intercept	2190.2	93.47	6	23.433	<0.001
Front (STF)	-745.4	104.49	6	-7.133	<0.001
Sex (Male)	82.4	104.49	6	0.788	0.461
**Daily travel distance [km/day]**					
Intercept	74.9	4.29	6	17.459	<0.001
Front (STF)	-11.9	4.80	6	-2.488	0.047
Sex (Male)	-1.6	4.80	6	-0.326	0.756
**Trip duration [days]**					
Intercept	69.1	4.59	2	15.066	0.004
Front (STF)	-0.4	6.49	2	-0.066	0.953
Sex (Male)	2.6	6.49	2	0.396	0.730
**Trip length [km]**					
Intercept	6156.7	478.54	2	12.866	0.006
Front (STF)	-1823.1	676.75	2	-2.703	0.114
Sex (Male)	-691.1	676.75	2	-1.021	0.415

* Transformed to Julian Date for analysis. Intercept value has been back-transformed for clarity.

### Habitat selection at trip destinations

**Fig 4:**
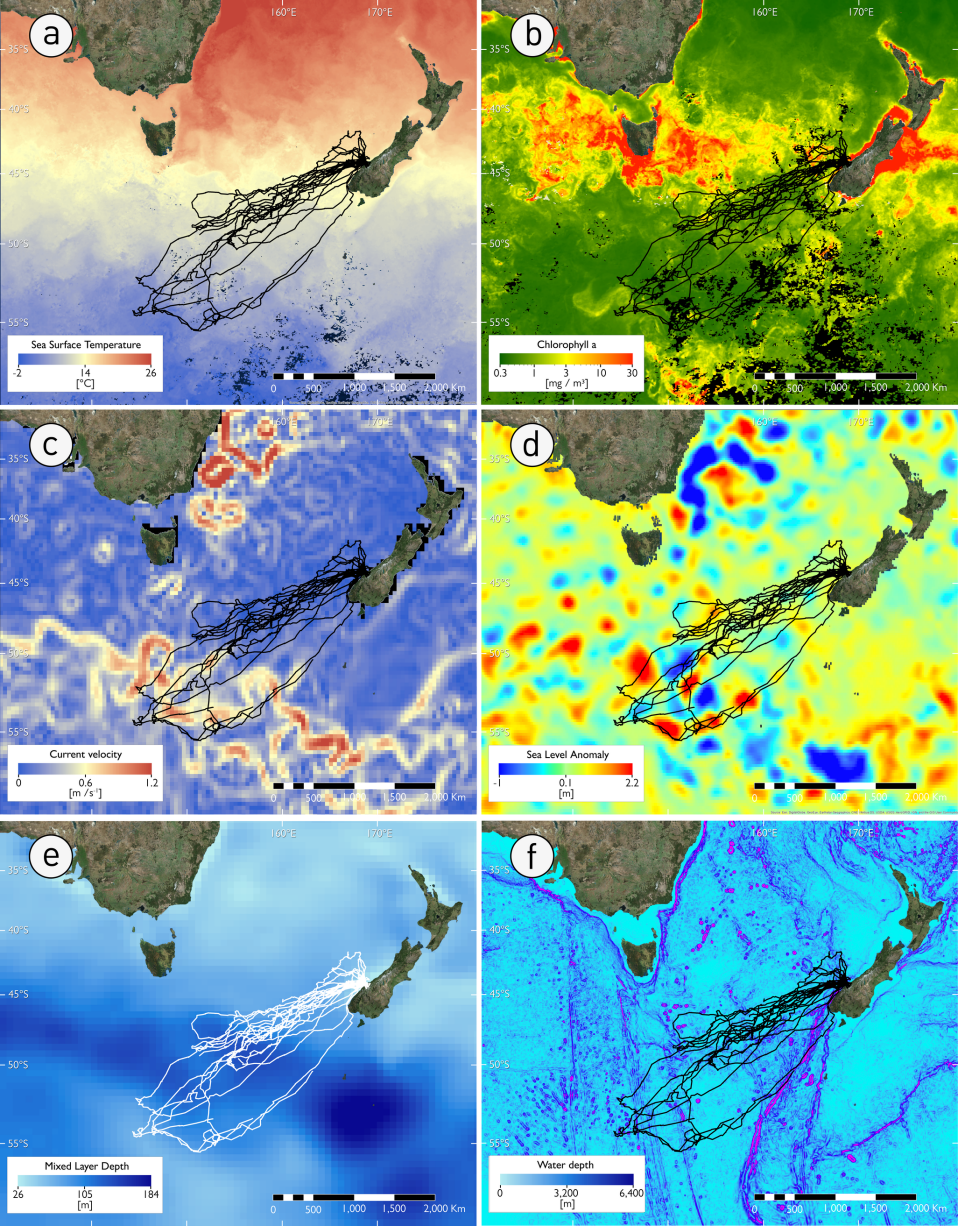
Tawaki pre-moult movements in relation to various environmental variables. a) Sea Surface Temperature (SST), b) Surface Chlorophyll a concentration, c) Surface current velocity, d) Sea Level Anomaly (SLA), e) Mixed Layer depth (MLD) and, f) Bathymetry slope. All variables represent rolling 32-composited centred around the median trip reversal date (04.01.2017), except MLD (see methods).

**Fig 5:**
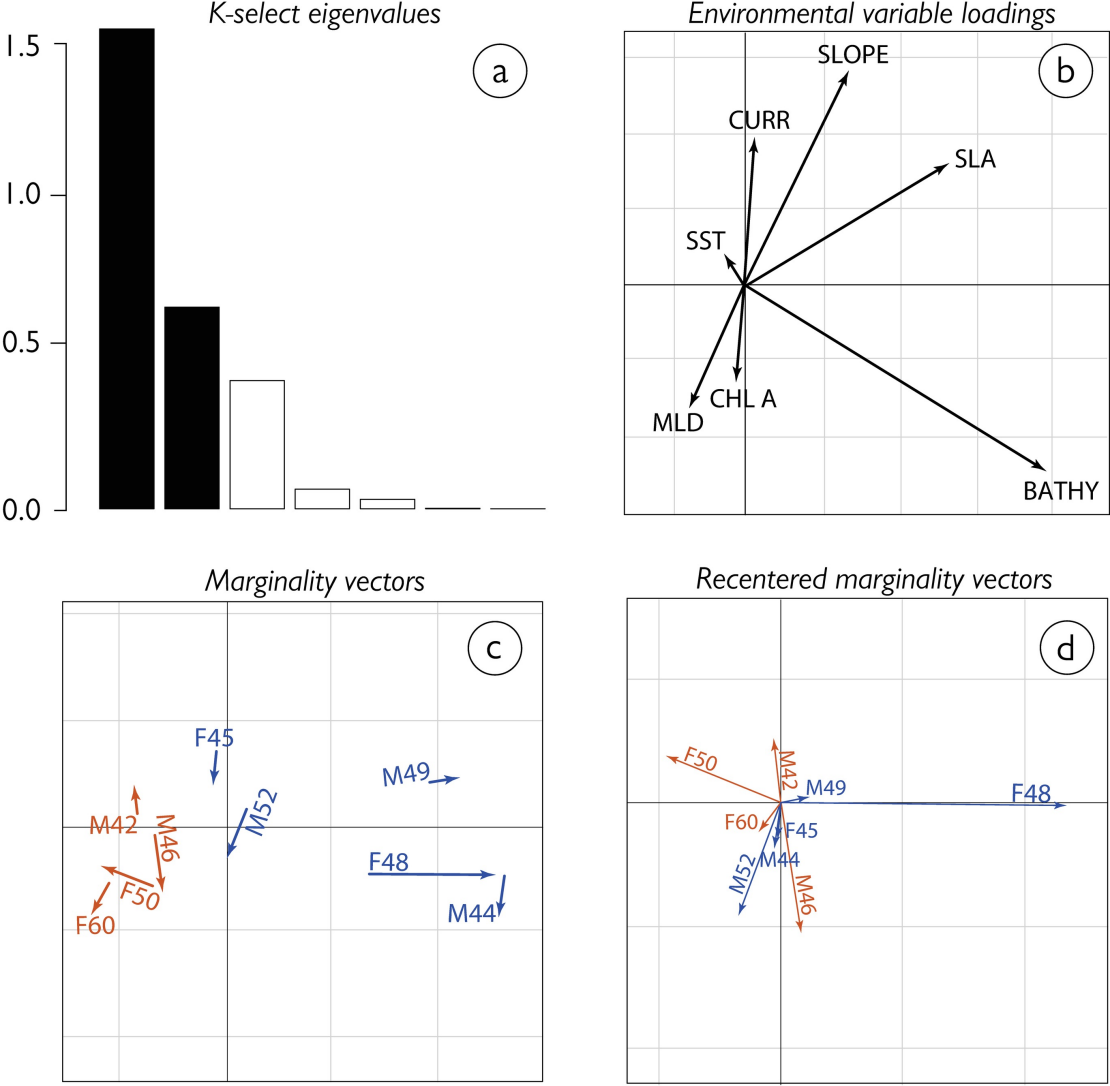
Habitat selection of nine Tawaki during two weeks centred around their trip reversal date, i.e. at their pre-moult journey destinations. (A) Bar chart of the K-select eigenvalues measuring the mean marginality explained by each factorial axis. (B) Loadings of environmental variables on the first two factorial axes–surface current velocity (CURR), seafloor sloping gradient (SLOPE), Sea Level Anomaly (SLA), water depth (BATHY), Chlorophyll a concentration (CHLA A), Mixed Layer Depth (MLD) and Sea Surface Temperature (SST). Note that water depth (BATHY) is measured as negative integers so that its loading is reversed in comparison with all other variables. (C) Marginality vectors of the individual penguins, where the base of the arrows indicates the mean composition of the habitat at the journey destination, while arrow length and direction correspond to the mean characteristics of the habitat the individuals moved towards during that time. (D) Recentered projection of the marginality vectors such that habitat availability is the same for all animals.

Satellite data of oceanographic features indicate differences at the two main trip destinations (Fig 4). This difference manifested also in the K-select analysis. Axis 1 and 2 accounted for 81% of the marginality in all individuals (Fig 5A) and were, thus, retained in the analysis. Penguins travelling to the STF selected waters with greater mixed layer depth and higher Chlorophyll a concentration (Fig 5C and 5D, orange arrows), whereas penguins at the SAF (Fig 5C and 5D, blue arrows) preferred waters with reduced depth, increased sea level anomaly, generally associated with increased surface currents.

## Discussion

### Device impact and reasons for cessation of transmission

Externally attached devices inevitably alter the hydrodynamics of penguins [36] which can negatively affect foraging parameters and success [37,38]. This is especially true for devices featuring an external antenna as used in this study which further increases drag [39]. To mitigate additional drag, devices were attached to the lower back [36] and coated smoothly with a layer of epoxy resin. Most devices ceased transmission before the penguins had completed their pre-moult journeys, for either technological or biological reasons. Devices failing to transmit shortly after device attachment probably had either technical failures or were a result of suboptimal attachment so that penguins were able to remove the units, a behaviour occasionally observed in penguins fitted with various types of external devices [40–42]. Similarly, cessation of transmission later on during the tracking period might have been due to device detachment. Obviously, the death of a study bird, e.g. predation or starvation, could also explain a premature end of transmission; the presence of the attached device could contribute to such an outcome. However, considering that the average deployment weight of birds that could be tracked until their return to the mainland was lower (mean body mass: 3,000±354 g, n = 5) than that of birds where transmission ceased prematurely (3,525±373 g, n = 12) device loss is the more likely explanation. Therefore, we assume that the transmitters were not detrimental to the penguins and have not essentially altered the birds’ behaviour.

### Spatial segregation of trip destinations

There were two distinct groups of Tawaki that foraged either at the Subtropical Front (STF) or the Subantarctic Front (SAF): Birds that started their pre-moult trips in the second half of November all foraged closer to the STF, whereas birds that left in first half of December tended to travel to the SAF. As birds used very similar outward-bound travel trajectories (Fig 2A), it appears likely that the choice of trip destination is determined by the individual circumstances (e.g. being a breeder or not) rather than environmental conditions encountered by the birds during their outward travel.

Marked sex-dependent differences in the choice of foraging habitat were reported during the pre-moult stage in Macaroni penguins from Crozet and Kerguelen [43] as well as Rockhopper penguins from Marion Island [44]. However, stable isotope analyses in Southern Rockhopper penguins from the Falkland Islands (Malvinas) showed that during the pre-moult stage both sexes forage on the same trophic levels indicating common foraging grounds [45]. This also applies to Tawaki–penguins of both sexes travelled either to the STF or SAF.

A possible explanation for this behaviour is that the choice of the trip destination is related to breeding status, which unfortunately we could not determine with certainty for birds fitted with PTTs. Breeding Tawaki usually depart on pre-moult trips after their chicks have fledged towards the end of November and early December [16]. Timing of breeding can vary between years and location [46], but is usually fairly synchronized in the respective colonies (egg laying occurs within a 10 day-period [16]). As the breeding areas still contained many chicks that had yet to finish their moult to juvenile plumage when PTT deployments were carried out, it can be assumed that fledging occurred in early December. As such it appears that the group of penguins that departed later and travelled to the SAF still engaged in chick provisioning at the time of deployment, whereas penguins visiting the STF may have been either non-breeders or failed breeders. This would correspond to observations by [16] that non-breeders depart colonies before breeding adults. Moreover, non-breeders are believed to return first to moult in late January and early February [16] which matches the return dates of birds that foraged at the STF (Table 1).

Spatial segregation of breeders and non-breeders during the pre-moult dispersal might be an indication of different dietary needs of birds in the two groups. Breeding is an energetically demanding period for birds as they have to balance the need for provisioning their offspring with their own sustenance [47,48]. Especially during the incubation and early chick-guard period, Tawaki spend long periods at the nest necessitating fasting spells of several weeks [16]. Moreover, prey targeted by penguins to feed their chicks can be different from the food more adequate for adult penguins [49]. Hence, it is plausible to assume that dietary needs of breeding birds that had limited access to food more suited for self-sustenance for more than two months, is different from the requirements of non-breeders that were not constrained by nesting duties.

### Environmental properties at trip destinations

The marine habitats in the two regions targeted by Tawaki during the pre-moult differed considerably. At the STF warm, salty, and micronutrient-rich waters from the subtropics meet cold, fresh, and macronutrient-rich subantarctic surface waters [50,51]. The resulting mixing processes at the front fuel high biological productivity [52]. The habitat selection analysis showed that penguins at the front preferred regions with increased surface Chlorophyll a concentration (Fig 5). In previous studies it has been shown that stable isotope ratios (δ^13^C) in penguin feathers, which provides information about prey consumption in the weeks prior to moult, correlate with increased Chlorophyll a concentrations, suggesting beneficial foraging conditions in regions of elevated phytoplankton biomass [53]. Greater volumes of zooplankton are generally associated with increased phytoplankton biomass [54].

The SAF separates Subantarctic Mode Water (SAMW), which is characterised by low stratification potential and increased transfer of nutrients, oxygen and CO_2_ into the interior ocean, and low saline Antarctic Intermediate Water (AIMW) which subducts at the SAF [55]. The SAF also represents the northern boundary of the Antarctic Circumpolar Current (ACC) which results in the formation of substantial mesoscale ocean features, i.e. eddies, that play an important role for the accumulation of biological productivity [56]. Penguins travelling to the SAF showed a preference for ocean regions with increased surface currents and generally lower water depths and increased slope gradients (Fig 5) due to the presence of sea mounts (Fig 1). These factors all support the formation of eddies which are clearly discernible as clusters of increased and reduced Sea Level Anomalies (Fig 4D). Similar mesoscale structures have been found to be important predictors for the pre-moult movements of other crested penguins [57–59].

How these different marine habitats are reflected in the penguins’ diet is difficult to assess. It has been argued that the physical properties of mesoscale features could facilitate the accumulation of macrozooplankton such as krill [58]. During the breeding season, squid and fish tend to play more important roles in the diet of Tawaki [60,61]. The coastal regions of New Zealand’s South Island are influenced by oceanographic processes in the STF which closely follows the continental shelf [62]. This suggests at a principal fish diet in penguins foraging at the STF. However, pending stable isotope analyses of feather samples taken from Tawaki over the past three years–including those fitted with satellite tags for this study–will provide more detailed insights into how spatial segregation during pre-moult trips is reflected in in the trophic niches the birds occupy.

Two birds exhibited almost congruent travel paths on their homeward journeys on which they followed the Macquarie Ridge (Fig 1) for more than 1,000 km towards the New Zealand mainland. The birds did not travel together but were one week apart, so that it can be assumed that external factors determined their congruent travel trajectories. In this case, it was likely the presence of the underwater ridge which influences local oceanographic processes [51,62] that the penguins can use for way finding [63].

### Comparison to other penguin species

To date, only a small number of studies have examined the foraging movements of penguins during their pre-moult dispersal. Emperor penguins travel up to 1,245 km away from the breeding colonies to moult [64], although it appears that shorter distances ranging around 500 km are more common in the species [65]. However, unlike Tawaki, Emperor penguins do not moult at their nest sites and do not return to their colonies before the commencement of the next breeding season [66]. Macaroni and Rockhopper penguins from Marion Island in the south-west Indian Ocean travelled only around 900 and 700 km away from their breeding colonies during the pre-moult stage, respectively [58]. Similar ranges were observed in Macaroni penguins from the Crozet and Kerguelen archipelagos in southern Indian Ocean [43]. Like in Tawaki, the destinations of those birds were major oceanic fronts. However, both species breed in localities that are located closer to these fronts.

### Biogeographical implications

Distances covered by Tawaki during the pre-moult phase are extraordinary and appear to be at the extreme of what a flightless, deep-diving bird species can accomplish in a reasonably short time. This raises a number of questions that in combination have substantial implications for the breeding distribution of the species.

Why do the Tawaki perform what must be energetically demanding journeys? The waters around the New Zealand mainland are highly productive especially during the summer months when the penguins undertake their pre-moult journeys [67]. Moreover, both Little and Yellow-eyed penguins, the other two penguin species endemic to the New Zealand mainland, are sedentary and remain in the vicinity of their breeding colonies all year round suggesting a stable diet situation even through the winter [68].

Perhaps the pre-moult destinations at two major subantarctic fronts of Tawaki are genetically pre-disposed? It has been suggested that broad migratory patterns in birds have a genetic basis [69–71], although this mechanism has recently been dismissed in dispersive seabird migration [72,73]. However, there are examples that show that such strategies may in fact be species and situation dependent [74–76]. Certainly, the consistency of travelling trajectories in Tawaki heading towards their pre-moult destination (Fig 2A) indicate far more goal-oriented, less dispersive migratory patterns.

Does Tawaki’s capability to travel such vast distances in a relatively short time indicate good body condition at the end of the breeding season compared to other crested penguin species? Rockhopper penguins are about the same body size and mass as Tawaki [7]. Rockhopper penguins leaving on their pre-moult journey from Marion Island weighed a mean 2.6±0.2 kg (n = 13 birds) [58]. In comparison, Tawaki in this study appear to have been substantially heavier (3.1±0.4 kg, n = 17). This might be an indication that the Gorge River penguins left in a better body condition than penguins from Marion Island. It suggests that the Tawaki experience better feeding conditions during the breeding season than Rockhopper penguins so that they leave for their pre-moult journey in better shape, allowing them to travel greater distances than the penguins from Marion Island. Conversely, however, this would mean that in seasons of poor foraging conditions off the New Zealand mainland, such long-distance travels might be detrimental for Tawaki survival.

Is the penguins’ apparent fixation on reaching subantarctic Frontal systems a limiting factor in their breeding distribution? The Tawaki from Gorge River breed towards the northern most extreme of the species’ breeding range at Heretaniwha Point, some 120 km further north [10]. Fossil records suggest that historically Tawaki may have been more wide-spread on New Zealand’s South Island [77,78]; a breeding attempt has even been reported from the North Island [79]. However, if that were the case the penguins would have to travel an additional 1,000 km to reach (and return from) the pre-moult travel destinations identified in this study. This would likely not be a viable migration scenario for the penguins under any circumstances. The location of the Subtropical Front in the Tasman Sea is believed to have shifted between 200 and 800 km southwards in the past 25,000 years [51]. Hence, while it seems plausible that the Tawaki population may have extended as far as the top of the South Island, it seems unlikely that the species ever gained a foothold on the North Island. To the east of New Zealand the STF is restricted to bathymetry and has not changed significantly [51]; hence, the fossil records from regions from the northern-eastern ranges of New Zealand’s South Island [77] likely originate from vagrant individuals rather than representing indications for a more wide-spread historical breeding distribution in New Zealand.

### Potential for fisheries interactions

**Fig 6:**
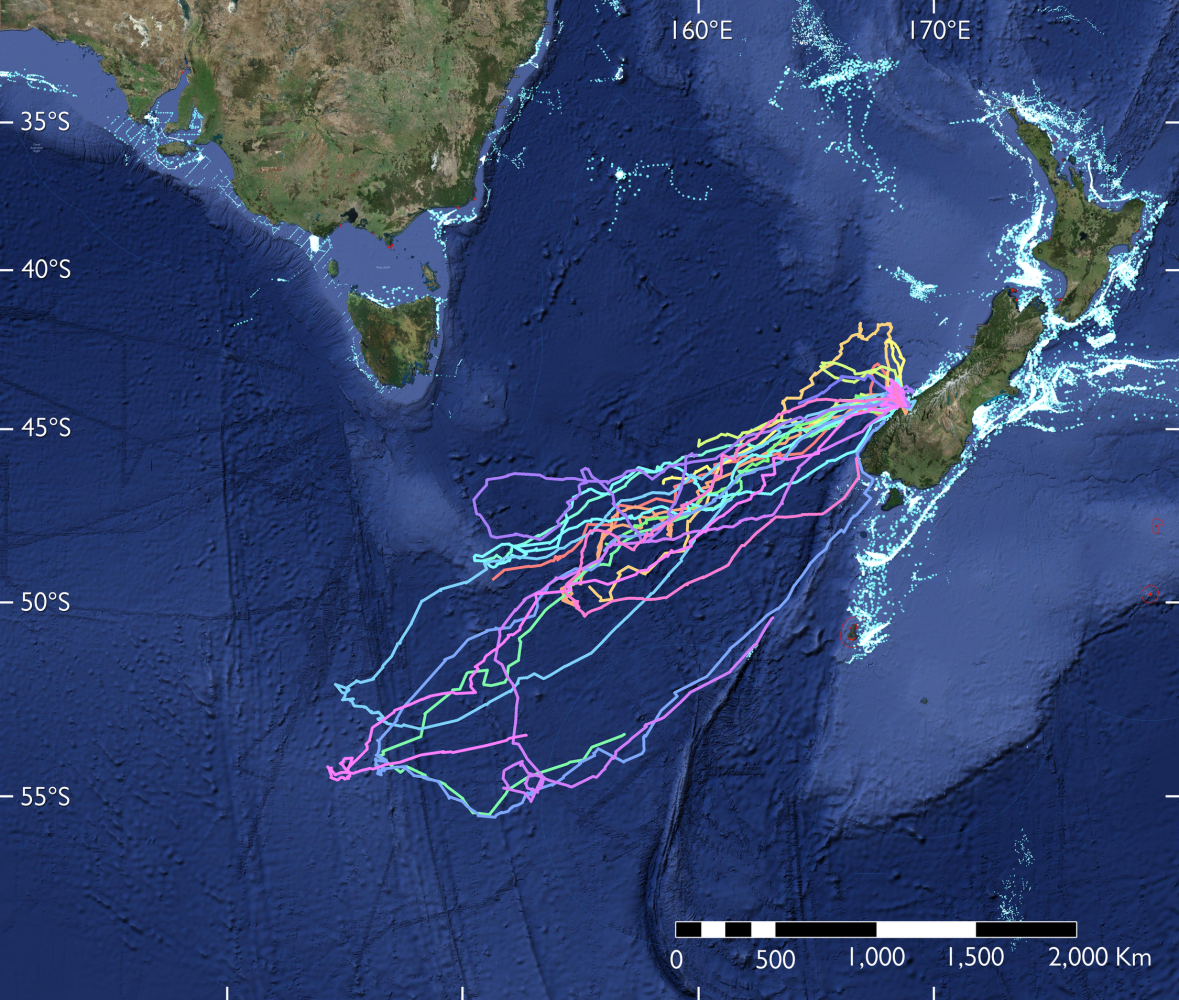
Commercial fishing activity in relation to Tawaki pre-moult dispersal. Light blue to white areas indicate regions with intense fisheries activities during the four months from November 2016 to February 2017. Fishing activity data were sourced from Global Fishing Watch (http://globalfishingwatch.org/map/).

The areas used by the tawaki penguins tracked in this study do not seem to overlap with areas of intense fishing effort in most part of the routes (http://globalfishingwatch.org/map/), except in the area immediately adjacent to the coast of New Zealand used by penguins when they leave and return to the mainland (Fig 6). Similarly, the southern Tasman Sea does not show a very high level of maritime traffic compared to other regions, although the penguin routes cross some navigation routes that link New Zealand with Australia (https://www.marinetraffic.com/en/ais/home/centerx:151.0/centery:-45.0/zoom:4). Hence, at least during their pre-moult migration Tawaki seem to face little to no risk from regional anthropogenic influences, unlike the other New Zealand mainland species [80,81]

## Conclusions

This study emphasizes the need to expand tracking of this species in the future, allowing determination of the width of the penguins’ migration corridor, the timing of movements, and the most critical areas for protection. In terms of adult survival one of the key demographic parameters for population developments [13,81], the pre-moult period is probably the most critical stage in the annual life-cycle in crested penguins. After an often energetically demanding breeding season, obtaining food quickly to build up resources for the similarly costly moult is crucial and renders the penguins particularly vulnerable to environmental perturbations [14]. Thus, monitoring of the pre-moult dispersal will provide vital information to understand the developments of key demographic parameters which drive population trends in this endemic, rare and threatened penguin species.
